# Coronarin D induces reactive oxygen species-mediated cell death in human nasopharyngeal cancer cells through inhibition of p38 MAPK and activation of JNK

**DOI:** 10.18632/oncotarget.22444

**Published:** 2017-11-14

**Authors:** Jui-Chieh Chen, Ming-Chang Hsieh, Shu-Hui Lin, Chia-Chieh Lin, Yi-Ting Hsi, Yu-Sheng Lo, Yi-Ching Chuang, Ming-Ju Hsieh, Mu-Kuan Chen

**Affiliations:** ^1^ Department of Biochemical Science and Technology, National Chiayi University, Chiayi, Taiwan; ^2^ School of Medical Laboratory and Biotechnology, Chung Shan Medical University, Taichung, Taiwan; ^3^ Department of Clinical Laboratory, Chung Shan Medical University Hospital, Taichung, Taiwan; ^4^ Department of Surgical Pathology, Changhua Christian Hospital, Changhua, Taiwan; ^5^ Cancer Research Center, Changhua Christian Hospital, Changhua, Taiwan; ^6^ Graduate Institute of Biomedical Sciences, China Medical University, Taichung, Taiwan; ^7^ Department of Otorhinolaryngology-Head and Neck Surgery, Changhua Christian Hospital, Changhua, Taiwan

**Keywords:** Coronarin D, nasopharyngeal carcinoma, apoptosis, autophagy

## Abstract

**Background and Purpose:**

Nasopharyngeal carcinoma (NPC) belongs to squamous cell carcinoma that occurs in the epithelial lining of the nasopharynx. Because of the anatomical position close to the cervical lymph node, some patients have a distant metastasis at the time of diagnosis that leads to treatment failure. Although early stages have a high curability and excellent prognosis, advanced NPC urgently requires new drugs developed to reinforce the effectiveness of therapy without noticeable side effects.

**Experimental approach:**

Coronarin D (CD), a natural product extracted from the rhizomes of *Hedychium coronarium*, has been reported to possess anticancer potential. The aim of the present study was to determine the anticancer activity of CD and further elucidate the underlying molecular mechanisms.

**Key Results:**

In this study, we first demonstrated that CD potently suppressed cell viability in various NPC cell lines. Treatment of cells with CD induced G2/M arrest, apoptosis, and autophagy. Further studies showed that CD increased the production of reactive oxygen species and subsequently activated both autophagy and apoptosis. Moreover, we found that CD-induced activation of p38 and JNK constituted major mechanisms involved in the apoptosis and autophagy triggered by CD. In particular, inhibition of autophagy could strengthen the cytotoxicity of CD, implying that autophagy seems to play a valuable survival and protective role in cancer cells.

**Conclusions & Implications:**

These findings provide a promise for the use of CD in combination with autophagy inhibitors for treatment of human NPC cell lines.

## INTRODUCTION

Nasopharyngeal carcinoma (NPC) is a type of head and neck cancer arising from the epithelial cells lining the nasopharynx. Genetic abnormalities and Epstein-Barr virus infection are implicated in the pathogenesis of the disease, which is more prevalent in Africa and Southeast Asia [1–3]. Due to the anatomical position of NPC close to the cervical lymph node, it is more likely to metastasize to other areas of the body, leading to difficulty in the use of surgery for treatment. Currently, the use of concomitant chemotherapy and radiotherapy has been shown to improve survival in patients with advanced NPC [4]. However, these therapies still produce severe adverse effects [5, 6]. Therefore, the development of new treatment modalities is urgently required to reduce side effects and to reinforce the effectiveness of therapy in NPC.

Phytochemicals are natural plant-derived compounds that have been verified to possess a wide range of biological activities including anticancer effects [7]. Phytochemicals are becoming more attractive for cancer prevention and treatment because they are proven to be an effective treatment with fewer side effects. Coronarin D (CD), a labdane-type diterpene, is isolated mainly from the rhizomes of *Hedychium coronarium* [8]. Previous studies have shown that CD possesses a variety of biological activities such as antiallergic activity [9], anti-inflammatory activity [10], antifungal activity [11], antimicrobial activity [12], and anticancer activity [13]. However, the anticancer effect of CD and its related mechanisms in nasopharyngeal cancer are still largely unknown.

Accumulating evidence suggests that phytochemicals act through multiple mechanisms to induce cell cycle arrest and cellular apoptosis, which exert their antitumor and chemotherapeutic effects [14, 15]. Recently, the role of autophagy in cancer therapy has also received extensive attention. Autophagy is a major intracellular degradation mechanism that promotes cell survival through the recycling of cytoplasmic organelles and proteins to provide an energy source and building blocks, leading to tumor cell survival [16]. However, the role of autophagy in cancer remains controversial. Growing bodies of evidence have described cell death associated with autophagic features in response to various antitumor agents [17, 18]. Thus, the intricate interplay between apoptosis and autophagy is considered to be two different death modalities that serve pivotal roles in the development of a therapeutic strategy for cancer treatment [19].

Many anticancer drugs can induce the generation of reactive oxygen species (ROS), which subsequently results in the disruption of mitochondrial membrane potential, leading to tumor cell apoptosis [20, 21]. Therefore, cancer treatment by means of enhancing intracellular ROS production may be considered an effective approach. However, numerous studies have shown that ROS levels contribute to autophagy activation in response to diverse anticancer drugs, suggesting that autophagy is involved in maintaining cellular homeostasis [22, 23]. Recent investigations have also shown that autophagy inhibition could enhance the efficacy of therapeutic strategies, inducing tumor cell death [24–26]. The aim of the present study was to investigate whether the cytotoxicity of CD on NPC cells is associated with intracellular ROS production and further elucidate the underlying mechanisms that involve cross talk between apoptosis and autophagy.

## RESULTS

### Cytotoxic effects of CD on human NPC cell lines

**Figure 1:**
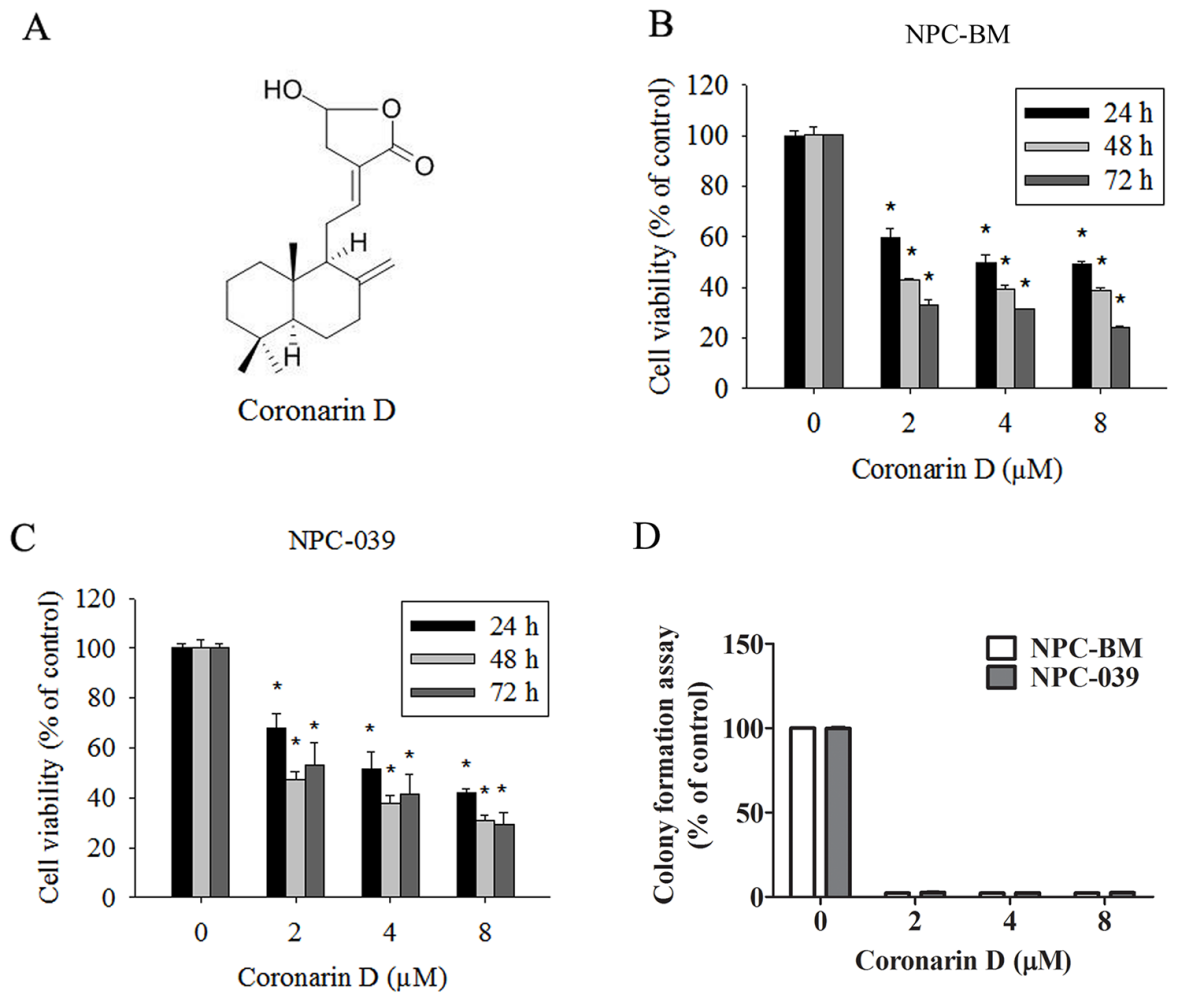
The dose- and time-dependent effects of Coronarin D (CD) on cell viability in human nasopharyngeal carcinoma (NPC) cells **(A)** Chemical structure of CD. **(B)** NPC-BM and **(C)** NPC-039 cells were treated with the indicated concentrations of CD (0-8 μM) for 24, 48 and 72 h. Cell viability was measured by MTT assay. **(D)** NPC-BM and NPC-039 cells cultured in condition medium presence of CD (0–8 μM) for 14 days, as analyzed by colony formation assay. Results are shown as mean ±SEM. from at least three independent experiments. ^*^P < 0.05, compared with the control (0 μM).

The chemical structure of CD is shown in Figure 1A. To examine the anticancer activity of CD on human NPC cells, NPC-BM and NPC-039 were treated with increasing concentrations of CD (0, 2, 4, and 8 μM) for 24, 48, and 72 h. As illustrated in Figure 1B and 1C, the viability of NPC cells markedly decreased in concentration- and time-dependent manners when compared with the control group (0 μM CD). The results as same as colony formation assay (Figure 1D). The cell viability decreased significantly after treatment with 8 μM CD for 24 h. Thus, all subsequent experiments were performed using 0–8 μm CD.

### CD-induced G2/M arrest and cell apoptosis in human NPC cell lines

**Figure 2:**
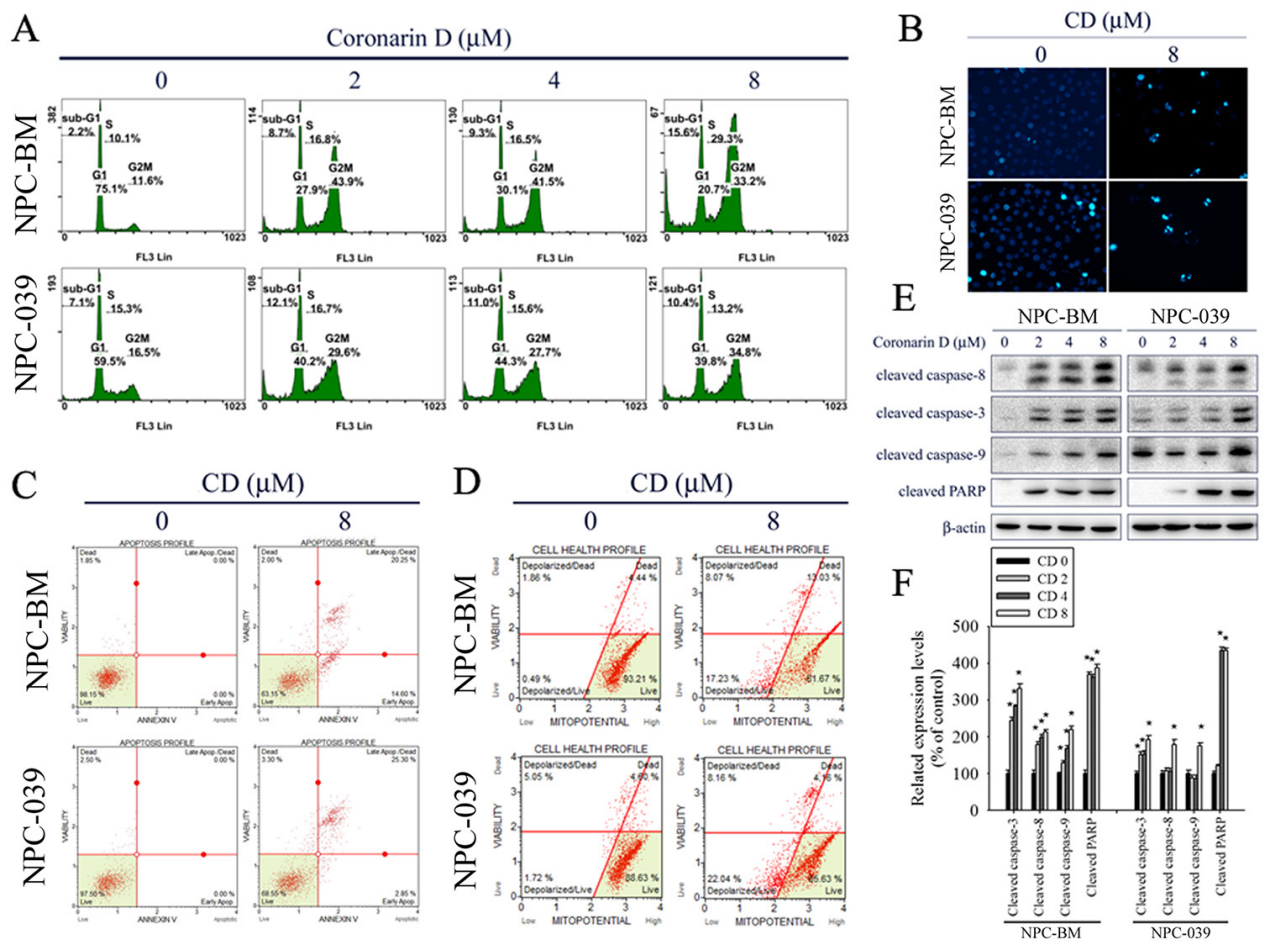
CD induces G2/M phase cell cycle arrest and apoptosis in NPC-BM and NPC-039 cells Cells were treated with indicated concentration of CD (0-8 μM) for 24 h. **(A)** PI staining and flow cytometry were performed to estimate cell cycle phase distribution (Sub-G1, G0/G1, S and G2/M). **(B)** Cells were fixed and stained with DAPI solution. Nuclear fragmentation and condensation were observed under a fluorescence microscope. **(C)** After treatment, cells were dual-stained with Annexin V-FITC/PI and analyzed by flow cytometry. **(D)** Effect of CD on mitochondrial membrane potential was done with Muse MitoPotential kit staining followed by flow cytometry. **(E)** A representative Western blot for expression of cleaved caspase-8, -3, -9 and PARP cleavage in cells were treated with increasing concentrations of CD. **(F)** Bar graphs represent the relative density of each band normalized to β-actin. Results are shown as mean ±SEM. ^*^P<0.05, compared with the control (0 μM).

To elucidate the cytotoxic mechanism of CD, the cell cycle distribution was analyzed by PI staining and flow cytometry. As shown in Figure 2A, CD increased cell numbers at the G2/M phase after treatment with increasing concentrations of CD for 24 h, accompanied by increased cell numbers at sub-G1 phases in NPC-BM and NPC-039 cells. We next explored the effects of CD on apoptosis in NPC cell lines. DAPI staining was used to estimate CD-induced changes in cell morphology. The results demonstrated an increasing number of apoptotic cells with condensed and fragmented nuclei after treatment with CD for 24 h (Figure 2B). To further quantify the extent of apoptosis, cells were double stained with annexin V-FITC/PI and subsequently analyzed by flow cytometry. As presented in Figure 2C, the percentages of cells demonstrating early stages of apoptosis (annexin V^+^/PI^−^) and late stages of apoptosis (annexin V^+^/PI^+^) were increased following treatment with CD at 8 μM for 24 h. To determine whether mitochondria-mediated pathways were involved in CD-induced apoptosis, we analyzed mitochondrial membrane potential levels by using a Muse MitoPotential Kit and Muse Cell Analyzer assays. The results showed that CD treatment caused an increase in the percentage of depolarized cells (Figure 2D), indicating that loss of mitochondrial membrane potential is involved in CD-induced apoptosis. To further clarify the type of apoptotic mechanisms induced by CD, the expression levels of cleaved forms of caspase-8, caspase-3, caspase-9, and PARP were investigated by Western blot analysis. The results revealed that CD significantly induced the formation of cleaved caspase-8, caspase-3, caspase-9, and PARP in a dose-dependent manner (Figure 2E and 2F).

### CD-induced autophagy in human NPC cell lines

**Figure 3:**
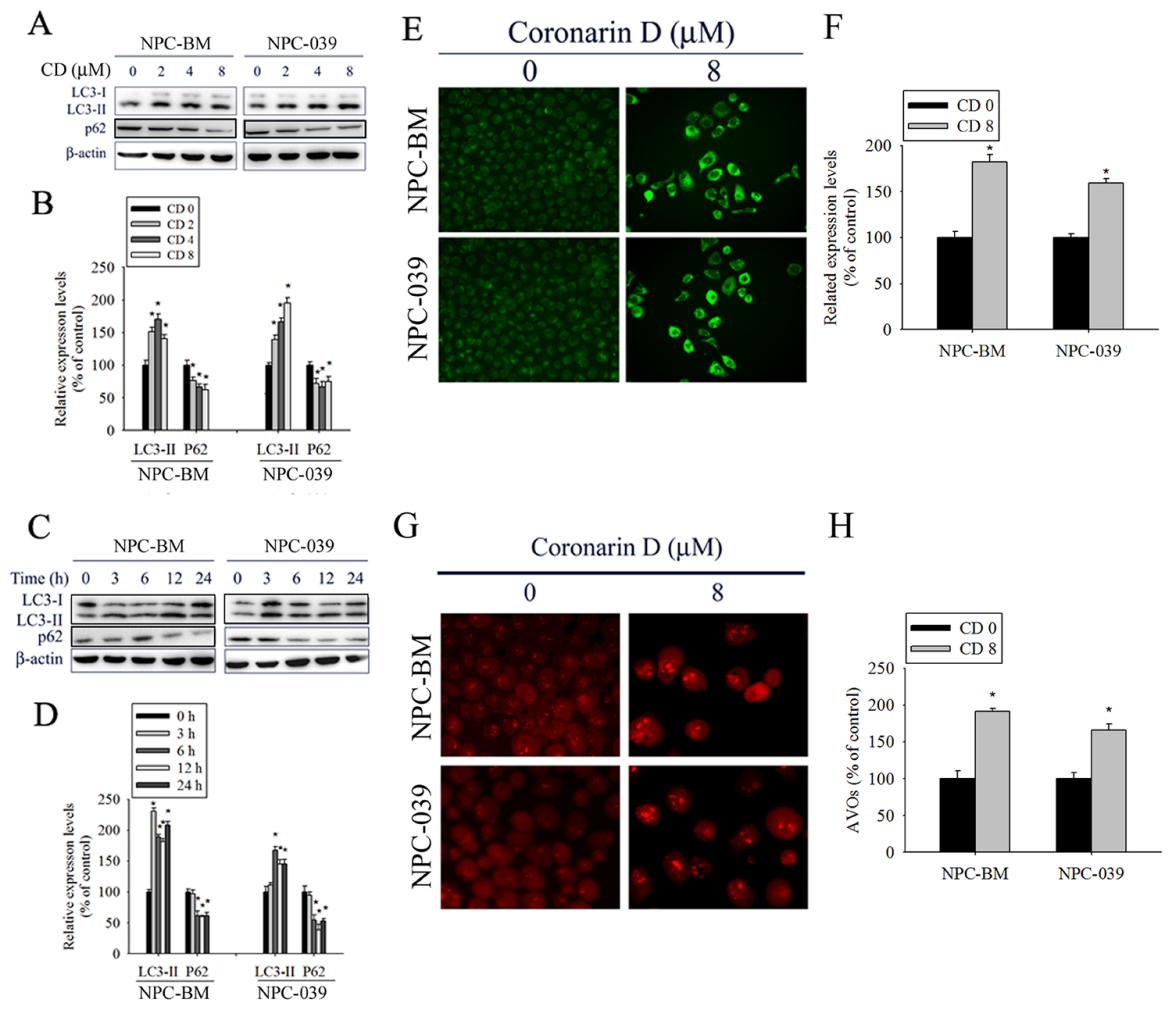
CD induces autophagy in NPC-BM and NPC-039 cells **(A)** Cells were treated with increasing concentration of CD (0-8 μM) for 24 h and the expression levels of LC3-I/LC3-II and p62 were examined by Western blot. **(B)** Bar graphs represent the relative density of each band normalized to β-actin. **(C)** Cells were incubated with CD (8 μM) for the indicated time intervals and the expression levels of LC3-I/LC3-II and p62 were examined by Western blot. **(D)** Bar graphs represent the relative density of each band normalized to β-actin. **(E** and **F)** Cells were treated with or without CD (8 μM) for 24 h, and then cells were stained with MDC fluorescent dye. MDC-stained cells were observed under a fluorescence microscope, which is an indicator of autophagosome formation. **(G** and **H)** Cells were treated with or without CD (8 μM) for 24 h, and then cells were stained with acridine orange (AO) for acidic vesicular organelles (AVOs) formation which was examined under a fluorescence microscope. The amount of AVOs (orange-red fluorescence) can be used as a marker of autophagosomes. Results are shown as mean ± SEM. ^*^P<0.05, compared with the control (0 μM).

Numerous studies have indicated that apoptosis and autophagy can both be activated by anticancer drugs. Thus, we investigated whether CD-induced apoptosis is accompanied by autophagy. First, we analyzed the effect of increasing concentrations of CD on the expression of autophagy-related proteins by using Western blot analysis. As expected, CD treatment increased the amount of LC3 II protein and reduced the expression of p62 in dose- and time-dependent manners, indicating a remarkable induction of autophagy (Figure 3A–3D). In addition, autophagy is characterized by increased formation of autophagic vacuoles. Hence, we examined the formation of autophagic vacuoles by using the specific fluorescent dyes MDC and AO. As shown in Figure 3E, the green fluorescence intensities of MDC-labeled autophagic vacuoles in CD-treated cells were much higher than those in the control. Consistently, cells treated with CD displayed a greater intensity of red fluorescence vesicles, AVOs, in the cytoplasm (Figure 3F). Taken together, these results clearly indicate that CD induces a robust autophagy in NPC cells.

### Role of ROS in CD-induced NPC apoptosis

**Figure 4:**
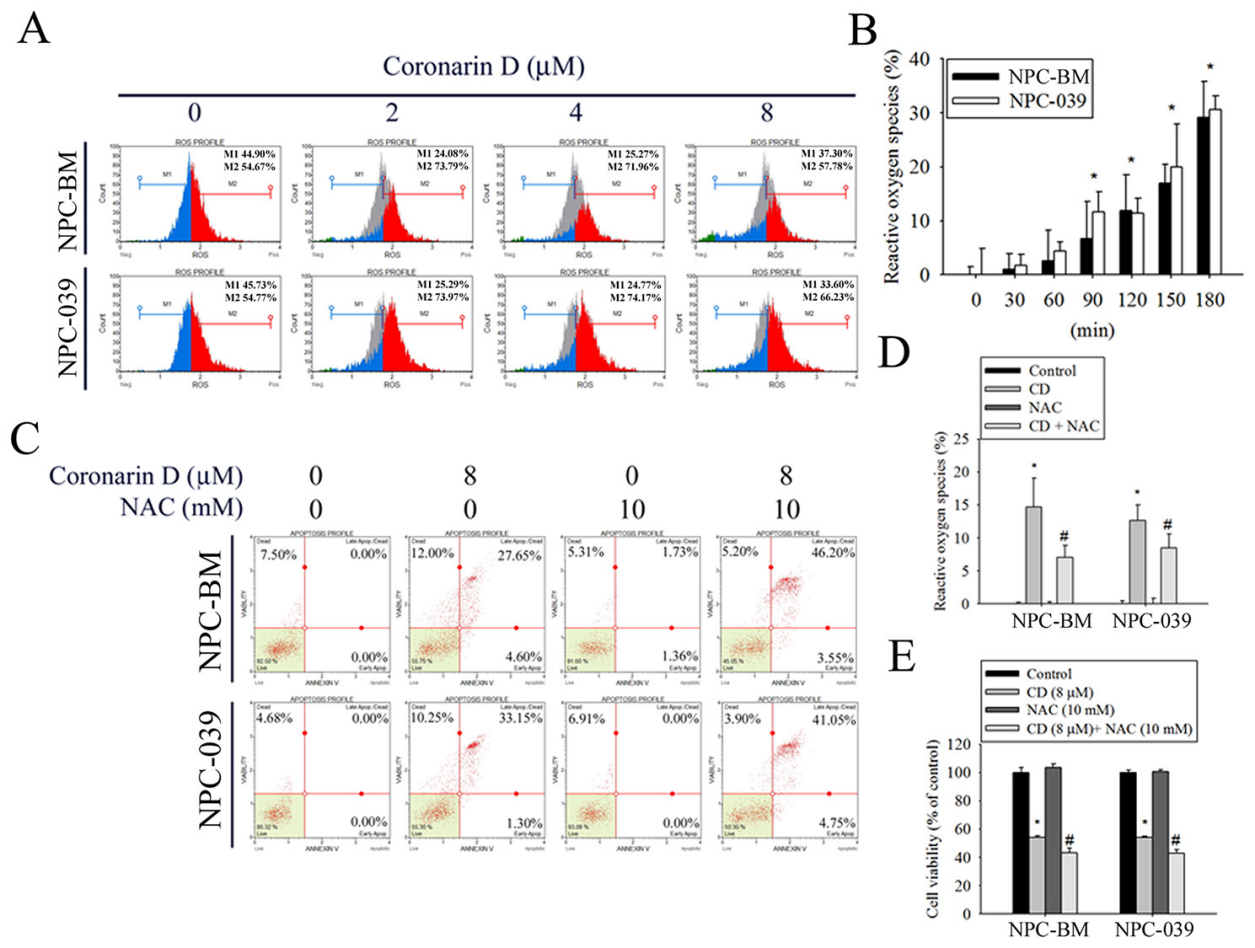
CD increases the generation of intracellular reactive oxygen species (ROS) to modulate cell death in NPC-BM and NPC-039 cells The amount of intracellular ROS was assessed using a DCFH-DA fluorescent probe and a flow cytometry. The green DCF fluorescence reflects intracellular ROS production. **(A)** Following treatment of cells with indicated concentrations of CD (0-8 μM) for 24 h, intracellular ROS generation was detected by flow cytometry using DCFH-DA staining. M1: negative control peak; M2: DCFH positive peak. **(B)** Percentage of green DCF fluorescence positive cells over total number of cells were calculated following 30, 60, 90, 120, 150, and 180 min exposure of 8 μM CD. **(C-E)** Cells were pretreated with or without 10 mM N-acetyl-L-cysteine (NAC; ROS scavenger) for 4 h, and then treated with or without 8 μM CD for 24 h. For quantitative analysis of apoptosis, cells were dual-labeled with PI and Annexin V fluorescence and analyzed by Muse Cell Analyzer flow cytometry (C). The scavenging ability of NAC for CD-induced ROS production was evaluated by percentage of green DCF fluorescence (D). Cell viability was determined by MTT assay (E). Data are representative of at least three independent experiments. Results are shown as mean ± SEM. ^*^P < 0.05, compared with the control (0 μM). ^#^*P* < 0.05, compared with cells treated with CD (8 μM).

Many studies have indicated that ROS serve as signaling molecules responsible for the modulation of anticancer drug-induced cell death to enhance therapeutic activity [27]. Therefore, the production of ROS was evaluated in CD-treated cells through DCHF-DA staining and flow cytometry. As shown in Figure 4A and 4B, exposure of cells to CD resulted in a dramatic increase in ROS production in dose- and time-dependent manners. Next, the ROS scavenger NAC was used to further characterize the relationship between ROS generation and cell apoptosis. Cell apoptosis was determined by annexin V/PI staining and flow cytometry analysis. Notably, the blockade of CD-induced ROS production by NAC pretreatment substantially enhanced CD-induced increases in late apoptotic cells (Figure 4C). As expected, NAC dramatically abolished CD-induced ROS generation (Figure 4D). Furthermore, the effect of NAC on CD-induced cell viability changes was detected using the MTT assay. The results displayed significantly reduced cell viability in the presence of NAC. Collectively, these results suggest that ROS inhibition enables the enhancement of CD-induced apoptosis.

### CD-induced ROS contributes to upregulation of NOX expression

**Figure 5:**
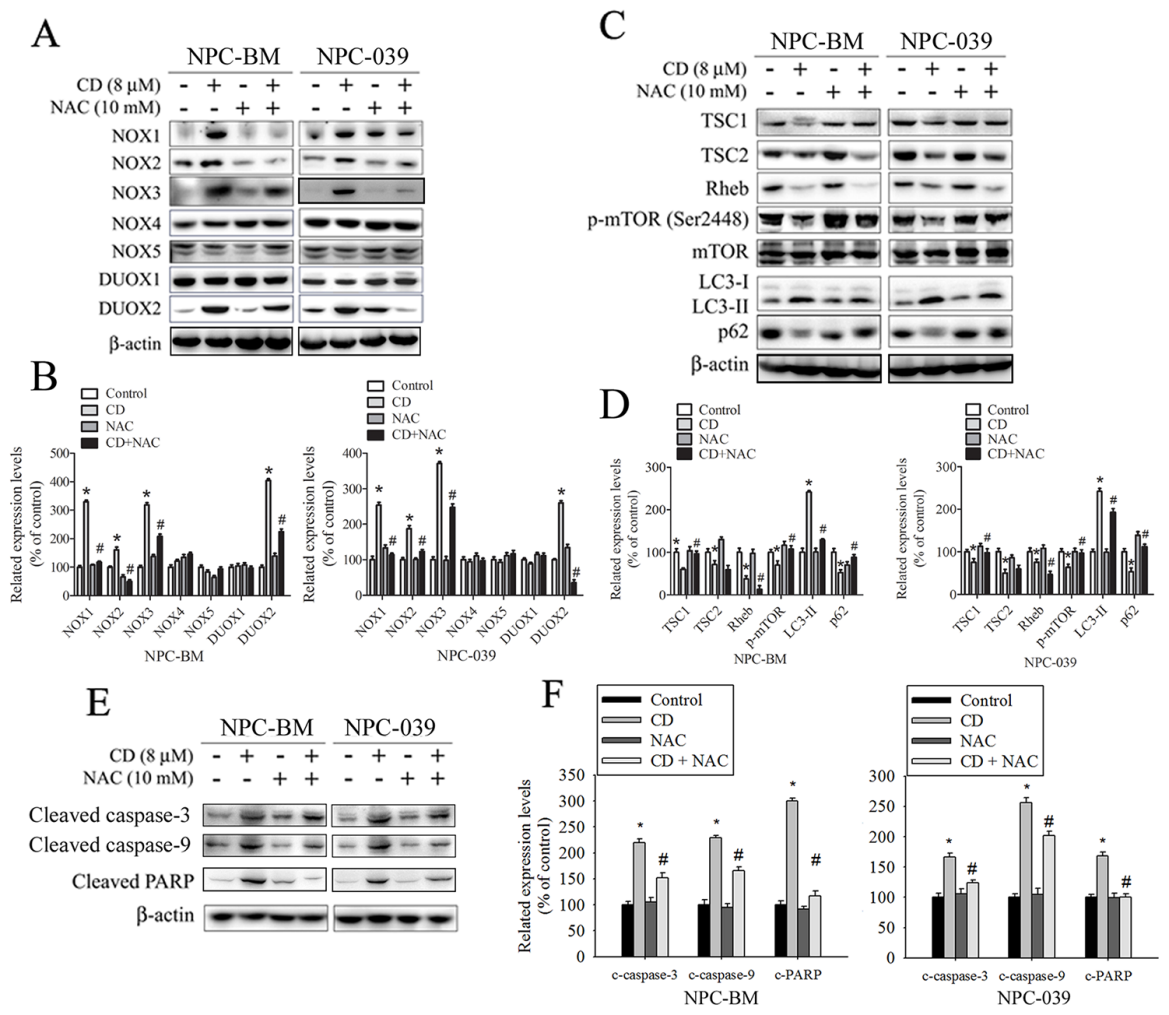
ROS is involved in CD-induced changes in expression of certain NOX/DUOX isoforms and modulation of TSC/Rheb/mTOR signaling axis to regulate autophagy and apoptosis in NPC-BM and NPC-039 cells Cells were pretreated with or without 10 mM NAC for 4 h, and then cultured with or without CD (8 μM) for 24 h. Equivalent amounts of total cell lysates were subjected to Western blot analysis. **(A)** NOX1, NOX2, NOX3, NOX4, NOX5, DUOX1, and DUOX2 were detected in cell lysates by Western blot. **(B)** The relative density of the bands was quantified by densitometry analysis. The histograms illustrate the relative expression levels after normalization to β-actin. **(C)** The TSC1, TSC2, Rheb, p-mTOR (Ser2448), mTOR, LC3-I, LC3-II, and p62 were analyzed by Western blot with their respective antibodies. **(D)** The histograms illustrate the relative expression of the indicated proteins normalized to β-actin. **(E)** Levels of cleaved caspase-3, cleaved caspase-9, and cleaved PARP were analyzed by Western blot. **(F)** The relative density of the bands is shown in the histogram. Values represent the mean ± SEM. from three determinations. ^*^P < 0.05, compared with the control (0 μM). ^#^*P* < 0.05, compared cells treated with CD (8 μM) alone.

NOX is a family of enzymes involved in ROS generation, which exhibits a close correlation with progression and cell death in a wide range of cancers [28, 29]. To address whether CD induction of ROS is associated with the activation of the NOX family, we exposed cells to NAC prior to stimulation with CD. Pretreatment of cells with NAC considerably blocked CD-stimulated NOX1, NOX2, NOX3, and DUOX2 expression, as assessed by Western blot analysis (Figure 5A and 5B). The finding indicates that CD induces ROS production, which in turn upregulates the expression of NOX family members, creating a positive feedback mechanism for ROS production.

### CD-induced autophagy and apoptosis are attenuated by treatment with NAC

To further understand the mechanism of NAC-enhanced CD-induced cell apoptosis, we studied whether increased ROS contributes to enhanced autophagy. Previous studies have indicated that the mammalian target of rapamycin (mTOR) complex is a negative regulator of autophagy, which can be activated by modulating TSC1, TSC2, and Rheb. In addition, TSC signaling nodes (TSC1, TSC2, and Rheb) can function as a cellular sensor for ROS [30, 31]. Thus, we hypothesized that ROS might play a role in the TSC/Rheb/mTOR signaling axis to engender autophagy activation after CD treatment. As shown in Figure 5C and 5D, treatment with CD led to a significant reduction in TSC1, TSC2, and Rheb expression, and consequently repressed mTOR activation. However, pretreatment of cells with NAC could significantly reverse the suppression of TSC1 expression and mTOR activation, but also noticeably enhanced the inhibition of Rheb expression. Consistent with these observations, administration of NAC reduced CD-induced autophagy in NPC cells, as assessed by Western blot analysis. Next, we investigated whether NAC is involved in CD-induced apoptosis in NPC cells. As illustrated in Figure 5E and 5F, NAC markedly attenuated CD-induced increases in the amount of cleaved caspase-3, cleaved caspase-9, and cleaved PARP. Taken together, these results demonstrate that NAC simultaneously reduces CD-induced autophagy and apoptosis, which regulates their balance and thereby partially promotes CD-induced cell death in NPC cells.

### AKT/ERK/p38/JNK signaling pathways are involved in CD-induced apoptosis and autophagy

**Figure 6:**
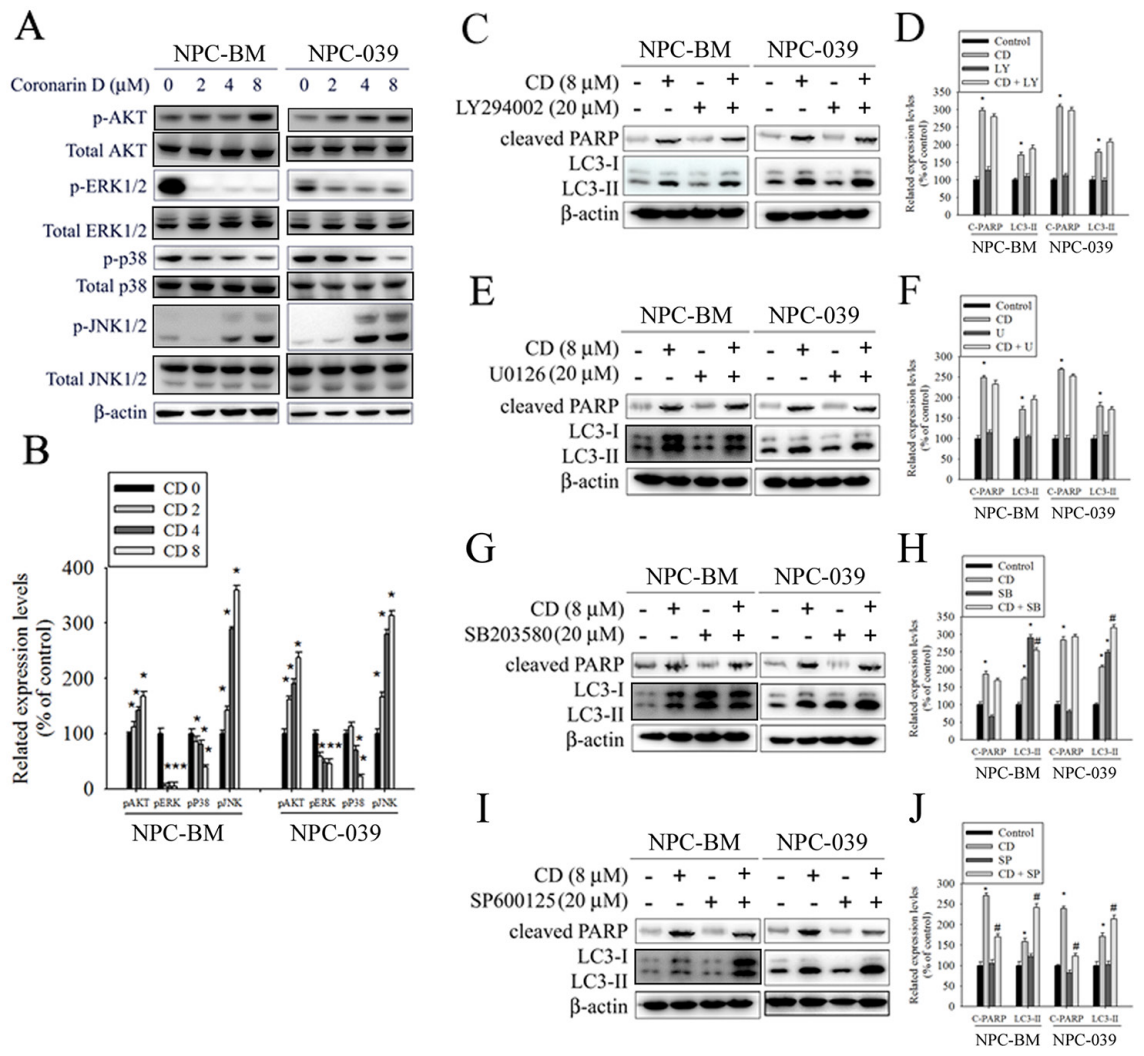
The effects of CD on activation of AKT and MAPKs in NPC-BM and NPC-039 cells **(A)** Cells were treated with increasing concentrations of CD (0-8 μM) for 24 h. Total cell lysates were analyzed by Western blot with specific antibodies against p-AKT, AKT, p-ERK1/2, ERK1/2, p-p38, p38, p-JNK1/2, p-JNK1/2, and β-actin. **(B)** The intensity of the phosphorylation signals normalized against their total protein levels. The histogram illustrates the relative phosphorylation levels. **(C-J)** Effects of the inhibition of AKT, ERK1/2, p38, and JNK1/2 on CD-induced apoptosis and autophagy were assessed by Western blot using specific antibodies (cleaved PARP and LC3-I/LC3-II). Cells were pretreated with LY294002 (AKT inhibitor, 20 μM), U0126 (ERK1/2 inhibitor, 10 μM), SB203580 (p38 MAPK inhibitor, 10 μM), or SP600125 (JNK inhibitor, 20 μM) for 1 h followed by treatment with or without CD for 24 h. The intensity of the band signals was quantified by densitometry and normalized to β-actin. The relative expression levels are shown in the histogram (right panel). Values represent the mean ± SEM. from three determinations. ^*^P < 0.05, compared with the control (0 μM). ^#^*P* < 0.05, compared cells treated with CD (8 μM) alone.

Previous studies have reported that the AKT and MAPK signaling pathways are implicated in apoptosis and autophagy [32–35]. Therefore, we evaluated the phosphorylation of AKT and MAPKs in response to CD exposure by using Western blot analysis. The results revealed that the stimulation of cells using CD induced an increase in the phosphorylation of AKT and JNK but engendered a reduction in the phosphorylation of ERK and p38 in a dose-dependent manner (Figure 6A and 6B). To further verify whether the AKT/ERK/p38/JNK signaling pathways are involved in CD-induced apoptosis and autophagy, cells were treated with an AKT inhibitor (LY294002), an ERK1/2 inhibitor (U0126), a p38 MAPK inhibitor (SB203580), or a JNK1/2 inhibitor (SP600125) for 2 h followed by treatment with or without CD for 24 h. The results showed that the AKT and ERK signaling pathways were not related to CD-induced apoptosis and autophagy (Figure 6C–6F). By contrast, pretreatment with SB203580 augmented the effects of CD on autophagy resulting from the conversion of LC3-I to LC3-II (Figure 6G and 6H). Moreover, pretreatment with SP600125 restrained CD-induced increases in cleaved PARP but enhanced the effects of CD on the levels of LC3II (Figure 6I and 6J). Taken together, our data suggest that both p38 and JNK are likely involved in the apoptosis and autophagy induced by CD.

### Effects of autophagy inhibition on CD-induced cell death in human NPC cell lines

**Figure 7:**
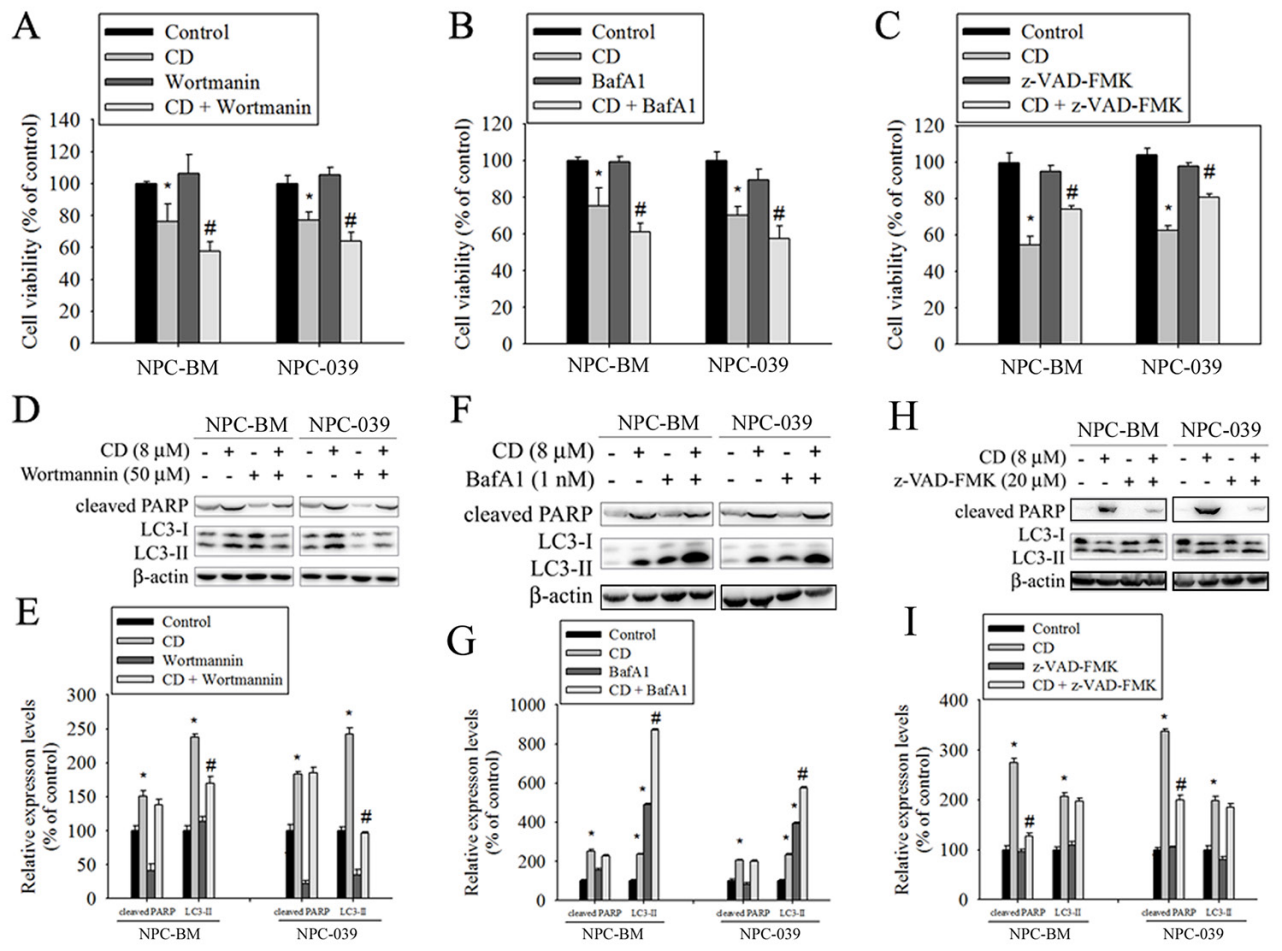
Inhibition of autophagy increased CD-induced cell death in NPC-BM and NPC-039 cells Cells were pretreated with Wortmannin (50 μM), BafA1 (1 nM), or Z-VAD-FMK (20 μM) for 1 h followed by treatment with or without CD for 24 h. **(A-C)** Cell viability was determined by MTT assay. **(D-I)** Crude lysates were prepared from NPC-BM and NPC-039 cells and analyzed by Western blot (upper panel) with antibodies against cleaved PARP and LC3-I/LC3-II. Beta actin was used as loading control. The respective protein levels were normalized to β-actin. The relative expression levels are shown in the histogram (lower panel). Values represent mean ± SEM. from three independent experiments. ^*^P < 0.05, compared with the control (0 μM). ^#^*P* < 0.05, compared cells treated with CD (8 μM) alone.

To clarify the relative roles of autophagy and apoptosis in CD-induced cell death, autophagy inhibitors (wortmannin and BafA1) and a broad-spectrum caspase inhibitor (Z-VAD-FMK) were used in the subsequent experiments. As revealed in Figure 7A and 7B, cotreatment of cells with CD and either wortmannin or BafA1 significantly enhanced cell death, compared with CD alone. However, CD combined with Z-VAD-FMK prevented reductions in cell viability in response to CD (Figure 7C). Next, we also measured the effects of inhibitors on CD-induced increases in the amount of cleaved PARP and LC3 II protein. The results showed that the level of LC3-II was effectively reduced in cotreatment with wortmannin (Figure 7D and 7E); however, co-treatment with BafA1 caused a remarkable accumulation of LC3-II (Figure 7F and 7G). BafA1 is known to be a strong inhibitor of the vacuolar type H^+^-ATPase and thereby inhibits the final step of lysosomal digestion in autophagy, and is able to block autophagy flux and increase cellular LC3-II levels. Both autophagy inhibitors did not change CD-induced increases in the formation of cleaved PARP. Moreover, cotreatment of cells with CD and Z-VAD-FMK effectively reduced CD-induced cleaved PARP levels but failed to inhibit an increase in the amount of LC3-II (Figure 7H and 7I). These data suggest that activation of autophagy is essential for cell survival under CD treatment.

## DISCUSSION

In the present study, we investigated whether CD exerts strong cytotoxicity against human nasopharyngeal cancer cells. This is the first report showing that CD can significantly promote G2/M arrest and the activation of both apoptosis and autophagy, leading to cell death in human NPC cells. We found that CD induced a marked increase in ROS generation, whereas pretreatment with the ROS inhibitor NAC remarkably attenuated the CD-induced activation of both apoptosis and autophagy. Moreover, the p38 and JNK signaling pathways were major mechanisms involved in the apoptosis and autophagy triggered by CD. We also examined the potential role of autophagy in CD-induced apoptotic cell death in human NPC cells. The results reveal that autophagy suppression can potentiate the cytotoxic activity of CD treatment, indicating the prosurvival function of autophagy to protect cells from death.

Intracellular ROS could originate from multiple sites such as the mitochondrial respiratory chain, cytochrome *P*-450 oxygenase, and NADPH oxidases [32]. ROS have been recognized as crucial intracellular signals involved in various physiological and pathological processes. Elevated ROS levels have been linked to the generation of genomic instability, thereby contributing to the process of carcinogenesis and cancer progression [33, 34]. However, emerging evidence indicates that natural anticancer compounds can induce cancer cell apoptosis by increasing ROS generation, which subsequently leads to the disruption of the mitochondrial membrane potential, resulting in tumor cell apoptosis [35, 36]. Therefore, anticancer agents by means of enhancing intracellular ROS production may be considered an effective approach.

The NOX/DUOX family of NADPH oxidases consists of seven members (NOX1-5 and DUOX1-2), and it has received increasing attention for its correlation with cancer development and progression [28, 37, 38]. In this study, we found that CD induced ROS generation, which in turn resulted in certain NOX/DUOX isoform (NOX1, NOX2, NOX3, and DUOX2) expression levels, which were abrogated by pretreatment with the ROS scavenger NAC. This finding indicates that some isoforms of NADPH oxidase can be activated from ROS, leading to further stimulation of ROS formation, which is a positive feed-forward ROS-induced ROS release mechanism by the NADPH oxidase axis to promote sustained amplification of ROS signaling. Consistent with our findings, previous studies have demonstrated that functional linkage between the mitochondria and the NOX family of NADPH oxidase is crucial for the sustained accumulation of ROS and cell death [38–41].

In addition, an emerging body of evidence indicates that ROS could be signaling molecules; they might induce autophagy to protect from death in response to a variety of therapeutic drugs [23, 42, 43]. Consistently, we found that inhibition of autophagy by pharmacological inhibitors (wortmannin and BafA1) significantly sensitized NPC cells to CD-induced cell death. The mTOR is a serine/threonine protein kinase that acts as a major negative regulator of autophagy. Rheb (Ras homolog enriched in the brain) is a small guanosine triphosphatase (GTPase) that is known as a key upstream activator of mTOR. The TSC 1/2 complex (tuberous sclerosis complex 1/2) is a GTPase-activating protein that inhibits the activity of Rheb by stimulating the conversion of Rheb-GTP to Rheb-GDP to repress mTOR signaling [44–46]. Hence, TSC1/2 inactivation can stimulate mTOR activation to inhibit autophagy. According to one report, the TSC signaling node is shown to serve as a cellular sensor for ROS to regulate mTOR and autophagy [30]. Our data demonstrate that CD promotes intracellular ROS generation, while simultaneously inhibiting TSC1, TSC2, and Rheb expression and repressing mTOR activation. Thus, the exact mechanisms through which CD inhibits mTOR activation via the TSC1/TSC2/Rheb axis require further exploration.

In conclusion, we demonstrated that CD induced significant G2/M arrest, apoptosis, and autophagy in human NPC cells. Furthermore, CD increased ROS generation and subsequently activated both autophagy and apoptosis. Although CD could activate the AKT/JNK pathways and suppress the ERK/p38 pathways, only the p38/JNK signaling pathways were involved in apoptosis and autophagy. In particular, inhibition of autophagy enhanced the cytotoxicity of CD, which suggests that a combination of CD and autophagy inhibitors may be a promising strategy for the treatment of human NPC cell lines.

## MATERIALS AND METHODS

### Chemicals

Coronarin D (purity > 95%) was purchased from ChemFaces (Wuhan, Hubei, China). It was dissolved in dimethyl sulfoxide (DMSO) and diluted with culture medium to the final concentration on the experimental day. The final concentration of DMSO for all treatments was consistently less than 0.1%. All cell culture reagents were purchased from Invitrogen (Carlsbad, CA, USA). The 3-(4, 5-dimethylthiazol-2-yl)-2, 5-diphenyltetrazolium bromide (MTT), propidium iodide (PI), RNase A, DAPI dye, protease inhibitor cocktail, phosphatase inhibitor cocktail, monodansylcadaverine (MDC), acridine orange (AO), and 2′, 7′-dichlorofluorescein diacetate (DCFH-DA) were obtained from Sigma-Aldrich (St Louis, MO, USA). Antibody against cleaved caspase-3, -8, and -9, cleaved poly (ADP-ribose) polymerase (PARP), LC3B, p62, NOX1, NOX2, NOX3, NOX4, NOX5, DUOX1, DUOX2 TSC1, TSC2, Rheb, p-mTOR (Ser2448), mTOR, p-AKT, AKT, p-ERK1/2, ERK1/2, p-p38, p38, p-JNK1/2, JNK1/2, and β-actin were purchased from Cell Signaling Technology (Danvers, MA, USA). Specific inhibitors for *N*-Acetyl-L-cysteine (NAC) AKT inhibitor (LY294002), ERK1/2 (U0126), p38 MAPK (SB203580), JNK (SP600125) Wortmannin and Bafilomycin A1 (Baf A1), and z-VAD-FMK were purchased from Santa Cruz Biotechnology (Santa Cruz, CA, USA).

### Cell culture

The human nasopharyngeal carcinoma (NPC) cell lines (NPC-BM and NPC-039) were a gift from Dr. Jen-Tsun Lin, Hematology & Oncology, Changhua Christian Hospital. All cells were cultured in RPMI 1640 medium supplemented with 10% fetal bovine serum (FBS), 0.1 mM non-essential amino acids, 1 mM glutamine, 1% penicillin/streptomycin (10,000 U/ml penicillin and 10 mg/ml streptomycin), 1.5 g/l sodium bicarbonate, and 1 mM sodium pyruvate (Sigma, St. Louis, MO, USA) and maintained at 37°C in a humidified atmosphere of 5% CO_2_.

### Cell cytotoxicity

Cells were seeded into 96-well plates at a density of 0.5 × 10^5^ cells/ml overnight. Afterwards, the cells were incubated with indicated CD concentration. At every indicated time interval after CD treatment, 20 μl of MTT (5 mg/ml) was added to each well and incubated for further 4 h at 37°C. The supernatant was then discarded, and 100 μl of DMSO was added to each well to dissolve the formazan crystals. Optical density (OD) was evaluated by measuring the absorbance, with a test wavelength of 490 nm and a reference wavelength of 630 nm.

### Colony formation assays

As previously described [47]. Human NPC-BM and NPC-039 cell lines were seeded at a concentration of 5×10^3^cells in 6-well cell culture plates in appropriate media. After 24h of incubation, media were replaced with fresh media containing CD at 2, 4, 8 μM. Incubates medium with compound changed every 3 days. Colonies were allowed to form for 2 weeks and then stained with 0.3% crystal violet solution. The supernatant was then discarded by DMSO. Optical density (OD) was evaluated by measuring the absorbance.

### Cell-cycle analysis

Cells were seeded (5 × 10^5^/well) on 6-well plates overnight and incubated with various concentrations of CD for 24 h. Afterwards, cells were fixed 70% ice-cold ethanol overnight, and then stained with PI buffer (4 mg/ml PI, 1% Triton X-100, 0.5 mg/ml RNase A in PBS) for 30 min in the dark at room temperature. The cell cycle distribution was analyzed by Muse Cell Analyzer (Millipore, Hayward, CA, USA).

### Detection of apoptosis

Apoptosis was detected though DNA condensation/fragmentation analysis using fluorescence microscopy (Lecia, Bensheim, Germany). Briefly, cells were collected and fixed by 4% paraformaldehyde for 20 min. Cells were then plated on the slides and stained with DAPI dye (50 μg/ml) for 10 min. The nuclear morphological changes related to apoptosis were assessed at least 500 cells. In addition, apoptosis was also analyzed by Annexin V and Dead Cell Assay (Merck Millipore, Germany). Briefly, cells (1 × 10^5^) were harvested and resuspended in 100 μl PBS with 2% BSA after treatment. Cells were then stained with Muse Annexin V & Dead Cell Reagent (100 μl) for 20 min at room temperature in the dark. The signals were measured Muse Cell Analyzer.

### Mitochondrial membrane potential assay

Cells were seeded on 6-well plates overnight and then treated with indicated concentrations of CD for 24 h. Then, cells were harvested with trypsin, washed with PBS and collected by centrifugation at 400 × g for 5 min. After centrifugation, the supernatant was removed and the cell pellets were stained with Muse MitoPotential dye for 20 min at 37°C. Cells were then incubated with 7-AAD, a dead cell marker, for an additional 5 min at room temperature. The data were analyzed by Muse Cell Analyzer (Millipore).

### Western blot analysis

Cells were harvested and lysed in RIPA buffer supplied with protease/phosphatase inhibitor cocktail. Protein concentration was determined by the BCA assay (Pierce). Equal amount of protein was separated by SDS-PAGE and transferred to PVDF membranes (Millipore, Bedford, MA). Membranes were blocked with 5% skim milk in PBS for 1 h and then incubated at 4 °C overnight with the indicated primary antibodies. Membranes were washed with PBS/0.1% Tween 20 (PBST) and incubated for 1 h at room temperature with the appropriate secondary antibodies conjugated to horseradish peroxidase. Membranes were then washed and bound antibodies were visualized using a chemiluminescence (ECL) detection kit (Millipore). The signals were examined and the relative photographic density was quantitated by AlphaImager 2000 systems (Alpha Innotech Corp., San Leandro, CA, USA).

### Detection of autophagic vacuoles

Cells were grown in 6-well plates followed by treatment with DMSO (vehicle) or indicated concentrations of CD for 24 h. Thereafter, cells were stained with 1 μg/ml AO or 50 μM MDC in fresh RPMI 1640 medium for 30 min at 37°C in the dark. After three times washing with PBS, cells were immediately visualized by a fluorescence microscope.

### Measurement of reactive oxygen species (ROS)

ROS was detected by using the florescent probe DCFH-DA which can be deacetylated to DCFH in the cells. The ROS levels were measured by dichlorofluorescein (DCF) which is an oxidized product of DCFH by ROS. Briefly, cells were exposed to the indicated dose of CD for 24 h, and then with stained with 25 μM of DCFH-DA for 30 min at 37°C in the dark. Cells were harvested and washed with PBS; intracellular ROS levels were evaluated by flow cytometer.

### Statistical analysis

Values represent the means ± standard deviation and the experiments were repeated at least three times. Statistical analyses were performed using the one-way analysis of variance (ANOVA) followed by Tukey's *post-hoc* test was used when more than three groups were analyzed. Data comparisons were performed with Student's *t* test (Sigma-Stat 2.0, Jandel Scientific, San Rafael, CA, USA) when two groups were compared. In all cases, a *P* value <0.05 was considered to be statistically significant.

## Abbreviations

NPC: nasopharyngeal carcinoma
CD: Coronarin D
ROS: reactive oxygen species
DMSO: dimethyl sulfoxide
FBS: fetal bovine serum
ERK1/2: extracellular signal-regulated protein kinases 1 and 2
JNK1/2: c-Jun N- terminal protein kinase 1 and 2
mTOR: mechanistic target of rapamycin
PARP: cleaved poly (ADP-ribose) polymerase
LC3: microtubule-associated protein 1A/1B-light chain 3
NOX: NADPH oxidase
DUOX: dual oxidase
TSC: tuberous sclerosis proteins
RHEB: Ras homolog enriched in brain

## References

[1] Jia WH, Qin HD (2012). Non-viral environmental risk factors for nasopharyngeal carcinoma: a systematic review. Semin Cancer Biol.

[2] Tu C, Zeng Z, Qi P, Li X, Yu Z, Guo C, Xiong F, Xiang B, Zhou M, Gong Z, Liao Q, Yu J, He Y (2017). Genome-wide analysis of eighteen Epstein-Barr viruses isolated from primary nasopharyngeal carcinoma biopsies. J Virol.

[3] Torre LA, Bray F, Siegel RL, Ferlay J, Lortet-Tieulent J, Jemal A (2015). Global cancer statistics, 2012. CA Cancer J Clin.

[4] Blanchard P, Lee A, Marguet S, Leclercq J, Ng WT, Ma J, Chan AT, Huang PY, Benhamou E, Zhu G, Chua DT, Chen Y, Mai HQ (2015). Chemotherapy and radiotherapy in nasopharyngeal carcinoma: an update of the MAC-NPC meta-analysis. Lancet Oncol.

[5] Kouloulias V, Thalassinou S, Platoni K, Zygogianni A, Kouvaris J, Antypas C, Efstathopoulos E, Nikolaos K (2013). The treatment outcome and radiation-induced toxicity for patients with head and neck carcinoma in the IMRT era: a systematic review with dosimetric and clinical parameters. Biomed Res Int.

[6] Yoshizaki T, Kondo S, Murono S, Endo K, Tsuji A, Nakanishi Y, Nakanishi S, Sugimoto H, Hatano M, Ueno T, Wakisaka N (2015). Progress and controversy for the role of chemotherapy in nasopharyngeal carcinoma. Jpn J Clin Oncol.

[7] Efferth T, Saeed ME, Mirghani E, Alim A, Yassin Z, Saeed E, Khalid HE, Daak S (2017). Integration of phytochemicals and phytotherapy into cancer precision medicine. Oncotarget.

[8] Chimnoi N, Pisutjaroenpong S, Ngiwsara L, Dechtrirut D, Chokchaichamnankit D, Khunnawutmanotham N, Mahidol C, Techasakul S (2008). Labdane diterpenes from the rhizomes of Hedychium coronarium. Nat Prod Res.

[9] Morikawa T, Matsuda H, Sakamoto Y, Ueda K, Yoshikawa M (2002). New farnesane-type sesquiterpenes, hedychiols A and B 8, 9-diacetate, and inhibitors of degranulation in RBL-2H3 cells from the rhizome of Hedychium coronarium. Chem Pharm Bull (Tokyo).

[10] Kiem PV, Thuy NT, Anh Hle T, Nhiem NX, Minh CV, Yen PH, Ban NK, Hang DT, Tai BH, Tuyen NV, Mathema VB, Koh YS, Kim YH (2011). Chemical constituents of the rhizomes of Hedychium coronarium and their inhibitory effect on the pro-inflammatory cytokines production LPS-stimulated in bone marrow-derived dendritic cells. Bioorg Med Chem Lett.

[11] Kaomongkolgit R, Jamdee K, Wongnoi S, Chimnoi N, Techasakul S (2012). Antifungal activity of coronarin D against Candida albicans. Oral Surg Oral Med Oral Pathol Oral Radiol.

[12] Reuk-ngam N, Chimnoi N, Khunnawutmanotham N, Techasakul S (2014). Antimicrobial activity of coronarin D and its synergistic potential with antibiotics. Biomed Res Int.

[13] Kunnumakkara AB, Ichikawa H, Anand P, Mohankumar CJ, Hema PS, Nair MS, Aggarwal BB, Coronarin D (2008). a labdane diterpene, inhibits both constitutive and inducible nuclear factor-kappa B pathway activation, leading to potentiation of apoptosis, inhibition of invasion, and suppression of osteoclastogenesis. Mol Cancer Ther.

[14] Surh YJ (2003). Cancer chemoprevention with dietary phytochemicals. Nat Rev Cancer.

[15] Priyadarsini RV, Nagini S (2012). Cancer chemoprevention by dietary phytochemicals: promises and pitfalls. Curr Pharm Biotechnol.

[16] Klionsky DJ (2007). Autophagy: from phenomenology to molecular understanding in less than a decade. Nat Rev Mol Cell Biol.

[17] Galluzzi L, Bravo-San Pedro JM, Levine B, Green DR, Kroemer G (2017). Pharmacological modulation of autophagy: therapeutic potential and persisting obstacles. Nat Rev Drug Discov.

[18] Chi KH, Wang YS, Huang YC, Chiang HC, Chi MS, Chi CH, Wang HE, Kao SJ (2016). Simultaneous activation and inhibition of autophagy sensitizes cancer cells to chemotherapy. Oncotarget.

[19] Qian HR, Shi ZQ, Zhu HP, Gu LH, Wang XF, Yang Y (2017). Interplay between apoptosis and autophagy in colorectal cancer. Oncotarget.

[20] Subramani R, Gonzalez E, Arumugam A, Nandy S, Gonzalez V, Medel J, Camacho F, Ortega A, Bonkoungou S, Narayan M, Dwivedi A, Lakshmanaswamy R (2016). Nimbolide inhibits pancreatic cancer growth and metastasis through ROS-mediated apoptosis and inhibition of epithelial-to-mesenchymal transition. Sci Rep.

[21] Zhang C, Jia X, Bao J, Chen S, Wang K, Zhang Y, Li P, Wan JB, Su H, Wang Y, Mei Z, He C (2016). Polyphyllin VII induces apoptosis in HepG2 cells through ROS-mediated mitochondrial dysfunction and MAPK pathways. BMC Complement Altern Med.

[22] Scherz-Shouval R, Elazar Z (2007). ROS, mitochondria and the regulation of autophagy. Trends Cell Biol.

[23] Dewaele M, Maes H, Agostinis P (2010). ROS-mediated mechanisms of autophagy stimulation and their relevance in cancer therapy. Autophagy.

[24] Amaravadi RK, Yu D, Lum JJ, Bui T, Christophorou MA, Evan GI, Thomas-Tikhonenko A, Thompson CB (2007). Autophagy inhibition enhances therapy-induced apoptosis in a Myc-induced model of lymphoma. J Clin Invest.

[25] Hu X, Shi S, Wang H, Yu X, Wang Q, Jiang S, Ju D, Ye L, Feng M (2017). Blocking autophagy improves the anti-tumor activity of afatinib in lung adenocarcinoma with activating EGFR mutations *in vitro* and *in vivo*. Sci Rep.

[26] Wiedmer T, Blank A, Pantasis S, Normand L, Bill R, Krebs P, Tschan MP, Marinoni I, Perren A (2017). Autophagy inhibition improves sunitinib efficacy in pancreatic neuroendocrine tumors via a lysosome-dependent mechanism. Mol Cancer Ther.

[27] Pelicano H, Carney D, Huang P (2004). ROS stress in cancer cells and therapeutic implications. Drug Resist Update.

[28] Roy K, Wu Y, Meitzler JL, Juhasz A, Liu H, Jiang G, Lu J, Antony S, Doroshow JH (2015). NADPH oxidases and cancer. Clin Sci (Lond).

[29] Lambeth JD, Krause KH, Clark RA (2008). NOX enzymes as novel targets for drug development. Semin Immunopathol.

[30] Zhang J, Kim J, Alexander A, Cai S, Tripathi DN, Dere R, Tee AR, Tait-Mulder J, Di Nardo A, Han JM, Kwiatkowski E, Dunlop EA, Dodd KM (2013). A tuberous sclerosis complex signalling node at the peroxisome regulates mTORC1 and autophagy in response to ROS. Nat Cell Biol.

[31] Pallichankandy S, Rahman A, Thayyullathil F, Galadari S (2015). ROS-dependent activation of autophagy is a critical mechanism for the induction of anti-glioma effect of sanguinarine. Free Radic Biol Med.

[32] Bae YS, Oh H, Rhee SG, Yoo YD (2011). Regulation of reactive oxygen species generation in cell signaling. Mol Cells.

[33] Juhasz A, Markel S, Gaur S, Liu H, Lu J, Jiang G, Wu X, Antony S, Wu Y, Melillo G, Meitzler JL, Haines DC, Butcher D (2017). NADPH oxidase 1 supports proliferation of colon cancer cells by modulating reactive oxygen species-dependent signal transduction. J Biol Chem.

[34] Lin XL, Yang L, Fu SW, Lin WF, Gao YJ, Chen HY, Ge ZZ (2017). Overexpression of NOX4 predicts poor prognosis and promotes tumor progression in human colorectal cancer. Oncotarget.

[35] Shen K, Xie J, Wang H, Zhang H, Yu M, Lu F, Tan H, Xu H (2015). Cambogin induces caspase-independent apoptosis through the ROS/JNK pathway and epigenetic regulation in breast cancer cells. Mol Cancer Ther.

[36] Zhang L, Fang Y, Xu XF, Jin DY (2017). Moscatilin induces apoptosis of pancreatic cancer cells via reactive oxygen species and the JNK/SAPK pathway. Mol Med Rep.

[37] Juhasz A, Ge Y, Markel S, Chiu A, Matsumoto L, van Balgooy J, Roy K, Doroshow JH (2009). Expression of NADPH oxidase homologues and accessory genes in human cancer cell lines, tumours and adjacent normal tissues. Free Radic Res.

[38] Skonieczna M, Hejmo T, Poterala-Hejmo A, Cieslar-Pobuda A, Buldak RJ (2017). NADPH oxidases: insights into selected functions and mechanisms of action in cancer and stem cells. Oxid Med Cell Longev.

[39] Dikalov S (2011). Cross talk between mitochondria and NADPH oxidases. Free Radic Biol Med.

[40] Zinkevich NS, Gutterman DD (2011). ROS-induced ROS release in vascular biology: redox-redox signaling. Am J Physiol Heart Circ Physiol.

[41] Kim YM, Kim SJ, Tatsunami R, Yamamura H, Fukai T, Ushio-Fukai M (2017). ROS-induced ROS release orchestrated by Nox4, Nox2, and mitochondria in VEGF signaling and angiogenesis. Am J Physiol Cell Physiol.

[42] Lee J, Giordano S, Zhang J (2012). Autophagy, mitochondria and oxidative stress: cross-talk and redox signalling. Biochem J.

[43] Poillet-Perez L, Despouy G, Delage-Mourroux R, Boyer-Guittaut M (2015). Interplay between ROS and autophagy in cancer cells, from tumor initiation to cancer therapy. Redox Biol.

[44] Inoki K, Li Y, Xu T, Guan KL (2003). Rheb GTPase is a direct target of TSC2 GAP activity and regulates mTOR signaling. Genes Dev.

[45] Guri Y, Hall MN (2016). mTOR signaling confers resistance to targeted cancer drugs. Trends Cancer.

[46] Kim LC, Cook RS, Chen J (2017). mTORC1 and mTORC2 in cancer and the tumor microenvironment. Oncogene.

[47] Hsieh MJ, Chen JC, Yang WE, Chien SY, Chen MK, Lo YS, Hsi YT, Chuang YC, Lin CC, Yang SF (2017). Dehydroandrographolide inhibits oral cancer cell migration and invasion through NF-kappaB-, AP-1-, and SP-1-modulated matrix metalloproteinase-2 inhibition. Biochem Pharmacol.

